# Pathogenesis and Treatment Options of Cancer Related Anemia: Perspective for a Targeted Mechanism-Based Approach

**DOI:** 10.3389/fphys.2018.01294

**Published:** 2018-09-20

**Authors:** Clelia Madeddu, Giulia Gramignano, Giorgio Astara, Roberto Demontis, Elisabetta Sanna, Vinicio Atzeni, Antonio Macciò

**Affiliations:** ^1^Department of Medical Sciences and Public Health, University of Cagliari, Cagliari, Italy; ^2^Medical Oncology Unit, “N.S. Bonaria” Hospital, San Gavino, Italy; ^3^Department of Medical Oncology, Azienda Ospedaliero Universitaria Cagliari, Cagliari, Italy; ^4^Department of Gynecologic Oncology, Azienda Ospedaliera Brotzu, Cagliari, Italy; ^5^Hospital Medical Management, Azienda Ospedaliera Brotzu, Cagliari, Italy

**Keywords:** cancer-related anemia, erythropoiesis, iron, inflammation, hepcidin, interleukin-6, leptin, erythropoiesis stimulating agents

## Abstract

Cancer-related anemia (CRA) is a common sign occurring in more than 30% of cancer patients at diagnosis before the initiation of antineoplastic therapy. CRA has a relevant influence on survival, disease progression, treatment efficacy, and the patients’ quality of life. It is more often detected in patients with advanced stage disease, where it represents a specific symptom of the neoplastic disease, as a consequence of chronic inflammation. In fact, CRA is characterized by biological and hematologic features that resemble those described in anemia associated to chronic inflammatory disease. Proinflammatory cytokine, mainly IL-6, which are released by both tumor and immune cells, play a pivotal action in CRA etiopathogenesis: they promote alterations in erythroid progenitor proliferation, erythropoietin (EPO) production, survival of circulating erythrocytes, iron balance, redox status, and energy metabolism, all of which can lead to anemia. The discovery of hepcidin allowed a greater knowledge of the relationships between immune cells, iron metabolism, and anemia in chronic inflammatory diseases. Additionally, chronic inflammation influences a compromised nutritional status, which in turn might induce or contribute to CRA. In the present review we examine the multifactorial pathogenesis of CRA discussing the main and novel mechanisms by which immune, nutritional, and metabolic components affect its onset and severity. Moreover, we analyze the status of the art and the perspective for the treatment of CRA. Notably, despite the high incidence and clinical relevance of CRA, controlled clinical studies testing the most appropriate treatment for CRA are scarce, and its management in clinical practice remains challenging. The present review may be useful to indicate the development of an effective approach based on a detailed assessment of all factors potentially involved in the pathogenesis of CRA. This mechanism-based approach is essential for clinicians to plan a safe, targeted, and successful therapy, thereby promoting a relevant amelioration of patients’ quality of life.

## Introduction

Anemia is a clinical status distinguished by a decreased erythrocyte mass with subsequent low hemoglobin (Hb) and hematocrit counts. The World Health Organization (WHO) and National Cancer Institute (NCI) have devised a scale to define anemia grade based on Hb values. As stated by WHO (accessed September/05/2017)^1^, normal Hb values are ≥12 g/dL in women, and ≥13 g/dL in men. The NCI grading of anemia is defined as follows: “mild (Grade 1), Hb from 10 g/dL to the lower normal limit; moderate (Grade 2), Hb 8.0–9.9 g/dL; severe (Grade 3), Hb <8 g/dL to 6.5 g/dl; life-threatening (Grade 4), Hb <6.5 g/dL”^2^.

Cancer-related anemia (CRA) is a sign that may accompany the evolution of cancer disease and is more commonly diagnosed in patients at advanced disease stages. It can occur independently from concurrent antineoplastic regimen, typically as a consequence of chronic inflammation associated to cancer disease. In fact, CRA biological and hematologic features resemble those described in anemia associated to chronic inflammatory disease. At this regard it should be specified that cancer patients with anemia should be sorted into two main categories: those with normal Hb values before starting medical treatment (often patients with limited, locally advanced resectable disease and candidate to undergo adjuvant cancer therapy) for whom anemia must be interpreted as a specific treatment-related toxicity (chemotherapy-induced anemia); and those with diagnosis of anemia preceding antineoplastic treatment (often receiving chemotherapy for advanced cancers). For the latter group, anemia is a mostly a result of the chronic inflammatory status existing in advanced neoplastic patients; this is the real CRA. This concept is fundamental to better understand the incidence and pathogenesis, and, therefore, the most appropriate treatment strategy for patients with CRA.

Cancer-related anemia is most often normochromic (MCH≥27 pg), normocytic (MCV between 80–100 fl) (Spivak, 2005; Adamson, 2008). Usually it is a hypoproliferative anemia with a reticulocyte count below normal (<25,000/microL) and a low value of reticulocyte index (normal range between 1 and 2), which is a more accurate measure of the reticulocyte count corrected against the severity of anemia on the basis of hematocrit (Rodgers et al., 2017). Additional features include normal/low serum iron concentrations (normal range 55–160 μg/dl for men and 40–155 μg/dl for women) and reduced total iron binding capacity (transferrin saturation <50%) (Rodgers et al., 2017), whilst ferritin values may be normal (30–500 ng/ml) or more often increased (≥500 ng/mL), with increased iron storage (Adamson, 2008). Hence, a defect in iron handling instead of a lack of iron, termed “functional iron deficiency,” has been hypothesized to underlie CRA. However, a low ratio of soluble transferrin receptor to ferritin could help distinguish CRA from iron deficiency-related anemia (Wish, 2006). Additionally, bone marrow erythroid hypoplasia is a feature of CRA and circulating erythropoietin (EPO) levels, the main erythrocyte growth factor, are inappropriately low in relation to the degree of anemia and intact renal function (Adamson, 2008).

The anemia prevalence rate in patients with cancer is remarkably high. Although anemia is commonly viewed as a toxicity related to antineoplastic chemotherapy, >30% of patients present with CRA at diagnosis before starting any antineoplastic treatments, rising to ∼67% once treatment is initiated (Ludwig et al., 2004; Birgegård et al., 2005). The prevalence of CRA is influenced by stage of disease (Caro et al., 2001; Knight et al., 2004; Birgegård et al., 2005): indeed, when we consider only patients with advanced cancer, CRA has been observed in a high percentage of men (77%) and women (68%) not undergoing chemotherapy (Dunn et al., 2003). Also advancing age may contribute to a higher incidence of anemia in cancer patients at diagnosis (Schwartz, 2007). Moreover, CRA prevalence differs among cancer types, with the highest percentage of anemic patients reported in lung cancer, gynecologic or genitourinary, and gastrointestinal tumors (Knight et al., 2004; Birgegård et al., 2005; Schwartz, 2007). At this regard a large, prospective, observational study carried out by our group in 888 neoplastic patients, at diagnosis before the implementation of any cancer treatments (Macciò et al., 2015c) showed that 63% of the patients had CRA, whose incidence increased with advanced cancer staging and decreased performance status (PS). We found that lung and ovarian cancer patients had the highest incidence (73.5 and 67.9%, respectively) and severity of CRA. Moreover, in our study Hb inversely correlated with the levels of inflammatory markers, hepcidin, ferritin, EPO, reactive oxygen species (ROS), and the modified Glasgow Prognostic Score (GPS). By contrast, Hb concentration was directly correlated with the levels of leptin, albumin, cholesterol, and antioxidant enzymes. These findings support the conclusion that CRA was a multifactorial inflammation-driven problem, with severity dependent on various components including the nutritional, energy metabolism, iron, and oxidative statuses.

## Clinical Relevance of CRA

Cancer-related anemia has a significant clinical impact in cancer patients: it is related with an important decline in PS and quality of life (QL), with progressive worsening of cognitive function and energy-activity levels (Cella, 1998; Crawford et al., 2002). Patients with CRA (Hb range, 8–10 g/dL) exhibit fatigue, lethargy, dyspnea, anorexia, and have difficulty concentrating, which can compromise their overall functional status and significantly reduce adherence to anticancer regimens (Ludwig and Strasser, 2001). In particularly, CRA-related fatigue may negatively influence patient QL and as a consequence the patient tolerance and motivation to sustain the antineoplastic regimen, thus not allowing patients to receive full and timely doses and potentially impairing the therapeutic response (Blackwell et al., 2004).

Moreover, CRA at diagnosis is a negative prognosticator for disease progression, survival (Obermair et al., 1998; Shin et al., 2014; Zhang et al., 2014; An et al., 2015) and overall death risk (Caro et al., 2001). CRA is also an established negative prognosticator for survival in early stage lung, breast, colorectal, and gynecological cancer patients candidate to surgery (Chamogeorgakis et al., 2008; Zhang et al., 2014; Cybulska et al., 2017). Additionally, CRA is associated with the decreasing efficacy of chemotherapy, radiotherapy, and chemoradiotherapy regimens, with a subsequent detrimental effect on patient prognosis (Thomas, 2001; Knight et al., 2004; Fuso et al., 2005; Gaspar et al., 2015; Zhu and Xu, 2015; Zhang et al., 2017). The relationship between CRA and reduced efficacy of chemo- and radiotherapy could be also a result of the increased aggressiveness of advanced neoplasia and the associated inflammation, which is known to affect prognosis and cause CRA (McMillan, 2009). However, experimental and clinical studies have shown that low oxygen (O_2_) levels (hypoxia) had a specific negative effect on the efficacy of antineoplastic treatment (Harrison and Blackwell, 2004; Cosse and Michiels, 2008; Wu et al., 2015; Xia et al., 2018). Cancer-related anemia could worsen tumor hypoxia, which in turn favors disease progression and metastases, and reduces tumor sensitivity to antiblastic treatments via various mechanisms including tissue acidosis, production of ROS, immunodepression, and alterations in tumor cells apoptosis (Höckel and Vaupel, 2001; Shasha, 2001).

Despite the prevalence of CRA and its significant clinical impact, its role at presently has been underestimated to such an extent that the indication for erythropoiesis stimulating agents (ESAs) remains limited to chemotherapy-related anemia (Rizzo et al., 2010; Rodgers et al., 2017). Therefore, specific targeted treatments for CRA have not yet been approved.

### Anemia-Related Symptoms

A major negative effect of anemia is the reduced capacity of erythrocytes to transport O_2_ around the body; therefore, affecting the metabolic activities and functional specificities of organ systems. Changes in cellular metabolism underlie the signs of anemia leading to compromised patient psychophysical well-being. Anemia symptoms characterize the compensatory mechanisms employed by the cardiovascular system and those subsequent to the decrease in Hb levels and reduced tissue oxygenation (Hare, 2014). O_2_ transport to the tissues is dependent on the following factors: erythrocyte Hb concentrations, overall blood Hb levels, O_2_ saturation of Hb, Hb-O_2_ dissociation curves, and tissue O_2_ tension. Changes in these parameters lead to compensatory mechanisms that not only induce the readjustment of the Hb-O_2_ dissociation curve but also involve the cardiovascular and renal systems (Woodson and Auerbach, 1982). The most important compensatory mechanism that attempts to balance the decreased capability of blood to transport O_2_ is initiated by the cardiovascular system to reduce peripheral vascular resistences and augment stroke volume thereby increasing heart output (Hare, 2014). On the other hand, the kidney reacts to hypoxia by increasing the production of EPO, a major regulator of erythropoiesis (Tsui et al., 2014). However, as mentioned earlier, based on the Hb levels, EPO levels are below the normal value in patients with cancer (Sanz Ortiz, 2008). Tissue hypoxia, causes cessation of glucose metabolism at the lactate level, preventing the conversion of lactate into pyruvate. Furthermore, lactate accumulation exerts a potent vasodilatory action that is strengthened by other vasoactive substances (e.g., bradykinin, adenosine, prostaglandin, nitric oxide, etc.), which are released from hypoxic tissues (Reglin et al., 2009). Consequently, the reduced efficacy of the vascular tone control systems increases blood flow to the periphery. Palpitations and sinus tachycardia are signs associated with an augmented cardiac output, whereas paleness, postural hypotension, and vertigo are correlated with the decrease in erythrocyte mass and lower vascular resistance in the peripheral circulation. Dyspnea, headache, sleep disorders, lethargy, depression, transitory cerebral ischemia, angina pectoris, limited functional capacity, and fatigue are caused by the lack of O_2_ availability in various organs and tissues. Anemia-related late signs include rest dyspnea, orthopnea, head vein distension, hepatomegaly, and edemas throughout the body (Pronzato, 2006).

Notably, in CRA, fatigue, weakness, and reduced physical and cognitive capacity are the most common symptoms, subsequent to the metabolic and psychological disturbances induced by tissue hypoxia (Miller et al., 2008; Boushel, 2017). Moreover, CRA has a significant impact on the central nervous system (CNS), particularly those sites more sensitive to hypoxia (Hare et al., 2008). EPO specific receptors are expressed in the CNS and EPO appears to exert a main role in preventing apoptosis and favoring the survival of neurons after a hypoxic, metabolic, or immunologic insult (Buemi et al., 2003).

Cancer-related anemia may strongly affects the immune system causing immunodepression (Bertero and Caligaris-Cappio, 1997), which increases susceptibility to infection and lowers antineoplastic efficacy. In fact, the metabolic damage subsequent to hypoxia is responsible for the lymphocyte functional deficit (Sitkovsky and Lukashev, 2005). Viceversa, recombinant human EPO (rhEPO) is able to augment the antineoplastic efficiency of T cells and humoral immunity (Katz et al., 2007; Nairz et al., 2012). Furthermore, treatment of patients with cancer using rhEPO increased T lymphocyte function regarding blastic response (Ghezzi and Mengozzi, 2007).

## Erythropoiesis

The comprehension of hematopoiesis, particularly erythropoiesis, and its regulatory processes is pivotal for understanding the pathogenesis of CRA, in order to apply the most pertinent and successful treatment choice. Through the erythropoiesis erythrocyte-programmed precursor cells continuously and neatly proliferate and differentiate into mature erythrocytes; thereby stabilizing or expanding the erythrocyte store as required. The rate of new cell production can be regulated by different physiological pathways that can also change under different pathological conditions (Doulatov et al., 2012). Erythrocytes have the main role to deliver O_2_ from the lungs to different body sites and CO_2_ in the reverse way. Considering that the basal O_2_ consumption is 4 mL/kg/min and the body O_2_ storage capacity is 20 mL/kg, it is essential to preserve an appropriate and steady erythrocyte mass, which, however, should be able at the same time to spread in response to tissue hypoxia (Benedik and Hamlin, 2014). In fact, under hypoxia, HIF-1 induces the synthesis of erythropoietin at molecular level (Jewell et al., 2001), alongside with vascular endothelial growth factor (VEGF), and several other growth factors to compensate for the negative consequences of low oxygen (Semenza, 2000). As a consequence, there is a log-linear increase in EPO levels that is inversely proportional to Hb or hamatocrit value (Spivak, 2005). Of note, O_2_ transported by Hb is essential for glucose metabolism and energy production; therefore, there is a close dependence between energy metabolism and O_2_ availability. On the other hand, the cellular O_2_ vehicle heme is synthesized from protoporphyrin IX (PPIX) as a product of glucose metabolism via the Krebs cycle. Therefore, although Hb is essential for O_2_ transport, glucose is indispensable for heme and, therefore, Hb synthesis. In this sense emerges the fundamental role of nutrients because without glucose and iron, heme synthesis cannot occur (Bennett and Kay, 1981). Erythropoiesis mainly depends from 4 distinct processes as follows: (1) proliferative potential of the erythroid progenitor reserve; (2) potency of the stimuli for erythrocyte production; (3) nutrient disposability (with an emphasis on the importance of glucose and iron); and (4) erythrocyte survival (which is reduced during hemorrhage or by early erythrocyte destruction) (Palis, 2014).

### Erythropoiesis and Iron

The differentiation phase of erythropoiesis from proerythroblast to erythrocyte is iron-dependent because of the need for heme and iron-sulfur clusters for the production of Hb. Heme is synthesized at an increased rate during erythroblast differentiation and, in turn, is needed to induce the *globin* gene (Doty et al., 2015).

The first stage of heme synthesis consists in the production of δ-aminolevulinic acid (ALA) through the condensation of succinyl-CoA and glycine in the mitochondrial matrix. This rate-limiting enzymatic step is mediated by 5-aminolevulinate (ALA) synthase 2, which is expressed exclusively in erythroid cells. ALA synthase 2 expression is significantly increased throughout the advanced phases of erythroid maturation, where it is fundamental for the terminal differentiation of erythrocytes. ALA synthase 2 expression is regulated by the presence in its gene of 5′ iron responsive element (IRE), which binds with IRE-binding proteins, thus connecting heme synthesis to iron. ALA is then transported in the cytoplasmic matrix and transformed in coproporphyrinogen III. Thereafter, coproporphyrinogen III inside the mitochondrial intermembrane space is transformed into protoporphyrinogen IX, which is then oxidized to PPIX. Finally, ferrous iron is incorporated, through a reaction catalyzed by ferrochelatase, an iron-sulfur cluster protein, to produce heme in the mitochondrial matrix; this reaction is another rate-limiting step in the heme biosynthesis process. The expression of ferrochelatase is iron-dependent and iron-sulfur cluster synthesis-dependent. Therefore, heme synthesis depends from iron intake by maturing erythroblast because iron is indispensable for the PPIX ring and also regulates the expression of ALA synthase 2 and ferrochelatase (Chiabrando et al., 2014). Considering the essential role of heme for Hb synthesis and erythropoiesis, it should be highlighted that a defect in the synthesis of ALA, which occurs in patients with inherited delta-aminolevulinic acid synthase 2 deficiency, leads sideroblastic anemia (Camaschella, 2008). Therefore, it could be hypothesized that metabolic alterations by affecting the integrity of glucose metabolism via the Krebs cycle and the related synthesis of the ALA precursor succinyl-CoA could negatively influence heme synthesis (McCammon et al., 2003), and then Hb levels (Oburoglu et al., 2016). Additionally, iron contributes to the regulation of EPO synthesis in the kidney through cross-talk with HIF-2-alpha. In detail, iron regulatory proteins bind to the iron responsive element of the kidney *HIF-2-alpha* gene modulating the translation of HIF and consequently, EPO expression. Moreover, an iron-dependent enzyme, prolyl-hydroxylase, catalyzes the degradation of HIF-2-alpha protein to the extent that is negatively related to the degree of hypoxia (Camaschella et al., 2016). Notably, the functional iron deficiency present in chronic inflammation-associated anemia and CRA negatively influences erythropoiesis in patients with advanced cancers. Similarly, it could be hypothesized that anorexia, associated to anemia, characterized by low glucose availability, could heavily interfere with adequate heme synthesis.

## Pathogenesis of Cancer Related Anemia

Cancer related anemia (CRA) refers to a condition occuring without bleeding, hemolysis, neoplastic bone marrow infiltration, kidney and/or hepatic failure. It principally results from the chronic inflammation associated with advanced stage cancer and the synthesis of proinflammatory cytokines by both immune and cancer cells (Weiss and Goodnough, 2005).

The main pathogenetic mechanisms by which inflammation may cause anemia include (Adamson, 2008):

• Shortened erythrocyte survival in conjunction with increased erythrocyte destruction
• Suppressed erythropoiesis in bone marrow
• Effects of inflammation on erythropoietin production
• Alterations in iron metabolism that result in iron-restricted erythropoiesis induced by hepcidin increase

The increased destruction of erythrocytes is mainly due to macrophage activation by different proinflammatory stimuli. The inhibition of erythropoiesis is related to two main mechanisms; iron restriction and direct inhibitory cytokine action on erythropoietic progenitors. Therefore, erythropoiesis is insufficient to compensate for the increased destruction of erythrocytes. Moreover, in patients with chronic inflammatory disease (as cancer) EPO shows a decreased synthesis in reply to hypoxic stimuli and its circulating concentrations are inadequately low for Hb levels, irrespective of intrinsic renal pathologies (Spivak, 2000). A direct effect of proinflammatory cytokines on kidney cells that produce EPO may contribute to the defective synthesis of this hormone (Jelkmann, 1998). A lot of evidence in the literature demonstrate that inflammation mediators exert a major contribution in the etiopathogenesis of CRA. In particular, proinflammatory cytokines (e.g., TNF-α, IL-1, IL-6), released by the cancer and activated immune cells in response to malignancy, may result in anemia by inducing changes to iron balance, inhibition of erythropoiesis, impairment of EPO synthesis and activity, reduction of erythrocytes lifespan and changes of energy metabolism (Means, 1995). Moreover, IL-1 and TNFα acts by activating the transcription factors GATA2 and nuclear factor-κB, both of which are negative regulators of the hypoxia-inducible factor 1 (HIF1) expression (Spivak, 2005).

Among cytokines, in particular IL-6 is able to induce hepatic synthesis of hepcidin, which regulates iron homeostasis by mediating the degradation of the iron export protein ferroportin 1, thereby inhibiting iron absorption from the small intestine and release of iron from macrophages. As a consequence iron is withdrawn from erythropoiesis (Ganz and Nemeth, 2011).

Moreover, chronic inflammation is correlated with increased concentrations of ROS (Macciò et al., 2005, 2015c), providing a partial explanation for the EPO deficit (Means, 2003). Indeed, ROS (O°⋅, H_2_O_2_, and OH^-^⋅) can inhibit EPO synthesis, by mimicking a false O_2_ signal in the renal peritubular interstitial cells. Oxidative stress can also increase the fragility of erythrocytes, decrease the amount of erythroid maturation, and reduce red cell survival (Sailaja et al., 2003; Olszewska et al., 2012; Lang et al., 2014; Bukowska et al., 2015). ROS also mediate the inhibitory effect of proinflammatory cytokines on erythroid precursor proliferation (Prince et al., 2012). Additionally, an *in vitro* study demonstrated that sustained H_2_O_2_ levels induce liver hepcidin expression through STAT-3 phosphorylation, by acting synergistically with IL-6, indicating another potential mechanism by which oxidative stress could contribute to CRA (Millonig et al., 2012).

Additionally, in advanced cancer patients, other triggering factors may contribute to anemia through a multifactorial pathogenesis; among them, the following mechanisms can be highlighted:

• Poor nutritional status
• Antineoplastic therapies (chemo- and radiotherapy) that may cause overt and/or aggravate CRA.

Of high relevance are in particular the metabolic and nutritional issues typical of advanced cancer patients and the defect of specific components (such as iron, vitamins, folic acid etc.) essential for erythropoiesis. Notably, the availability of these nutrients (e.g., glucose, iron) influence the synthesis of heme, which also depends on the efficiency of glucose metabolism via the Krebs cycle, and is essential, in combination with iron, for the synthesis of Hb (Chiabrando et al., 2014).

## Chronic Inflammation in Advanced Cancer Patients

During its development, the neoplastic disease is characterized by immunological alterations, which profoundly influence the patient clinical conditions potentially causing patient’s death (cancer cachexia syndrome) (Macciò and Madeddu, 2012; Argilés et al., 2014). In addition to anemia, the immune changes induce different symptoms involving several organs and processes: anorexia, nausea/vomiting, weight loss (with depletion of muscle and fat mass), raised energy metabolism with alterations of glucose, lipid and protein metabolism, fatigue, and immunosuppression (causing greater susceptibility to infections).

Although it is difficult to determine the precise onset of these changes, it has been established that they are consequent to the interplay between cancer and patient immune system (Delano and Moldawer, 2006). Activated macrophages, lymphocytes, and mesenchymal cells produce various soluble factors (cytokines) which are able to activate or inhibit different cells. In particular, IL-1, IL-6, and TNF-α are the principal factors involved in the cell-mediated immune response and also act as second transmitters for the synthesis of IL-2, which play a pivotal role in the control of the anticancer immune response. Some studies from our group have shown that the lymphocyte blastic response to various mitogenic stimuli, including phytohemagglutinin (PHA) and anti-CD3, is impaired in advanced cancer patients (Macciò et al., 1998; Mantovani et al., 2003). The intensity of the immune defect is proportional to cancer stage (Ladányi et al., 2004) and to the amount of inflammatory cytokines, in particularly IL-6, and other acute phase proteins (e.g., CRP) (Macciò et al., 1998). Indeed, the impaired lymphocyte functions should be assumed as a proxy of multiple functional changes, among which extremely important are the immunosuppressive action of macrophage-derived cytokines and the changes in energy metabolism, which are able to induce a status of oxidative stress. In our previous papers we showed that cancer patients are highly subjected to a state of increased oxidative stress (Macciò et al., 2009, 2015c; Madeddu et al., 2014). Notably, inflammatory cytokines not only have specific modulatory immune functions but are involved in the pathogenesis of the main metabolic changes and symptomatic aspects of the neoplastic patient (e.g., body weight loss and cachexia with reduction of muscle mass, anorexia, nausea/vomiting, etc.), thus influencing significantly patient’s well-being (Macciò and Madeddu, 2012). Therefore, this behavior of the immune system confers to the cancer the characteristics of a real chronic inflammatory disease with the related complications, such as anemia. In this regard, macrophages seem to be primarily involved in these events.

### Specific Actions of Proinflammatory Cytokines

It has been widely demonstrated that proinflammatory cytokines, particularly IL-1, TNF-α and IL-6 may affect erythropoiesis by the induction of: reduced proliferative response of erythroid progenitors; increased erythrocyte destruction by macrophages; and diminished response of erythroid precursors to EPO (Spivak, 2005). Moreover, the chronic activity of these same cytokines is mainly responsible of the multiple metabolic and nutritional changes occurring in advanced cancer patients (Macciò and Madeddu, 2012). Notably, by impairing the energy metabolism and the nutritional status, proinflammatory cytokines contribute to the pathogenesis of CRA.

Considering in detail the action of each cytokine, IL-1 is also able to inhibit erythropoiesis through directly and selectively suppressing replication and maturation of erythroid (BFU-e and CFU-e) precursors, reducing EPO receptor expression, and impairing EPO synthesis (Faquin et al., 1992; Jelkmann et al., 1992). Moreover, IL-1, together with other proinflammatory cytokines, has been implicated in activating macrophages for erythrophagocytosis, thus inducing a premature destruction and reduced survival of erythrocytes.

Moreover, IL-1 is involved in inducing several changes in energy metabolism and nutritional status. IL-1 induces anorexia associated with reduced food intake by acting in the hypothalamic nuclei of the CNS, where it inhibits the orexigenic factor Neuropeptide Y and indirectly increases the corticotropin releasing factor (CRF) levels (Patra and Arora, 2012); additionally, it stimulates CRF and somatostatin secretion, mediated by prostaglandin E2 (PGE2) (Gautron and Layé, 2010). This results in the inhibition of growth hormone (GH) accompanied by the subsequent reduction in the production of insulin-like growth factor-1 (IGF-1) that, in turn, cause muscle mass reduction typical of advanced cancer patients (Saini et al., 2006). Moreover, IL-1 inhibits the synthesis of insulin by pancreatic beta cells leading to hyperinsulinemia and insulin resistance (Burke et al., 2015). These IL-1 mediated activities may concur to the onset of CRA in advanced cancer patients. In particular, low glucose availability and insulin resistance negatively affect erythropoiesis since commitment into the differentiation stages is strictly dependent on glucose metabolism (Oburoglu et al., 2016). Erythroid differentiation is critically dependent on glucose cell uptake and glucose entry into the tricarboxylic acid (TCA) cycle that allows a high energetic yield for cell proliferation (Vander Heiden et al., 2009) as well as on glutamine-dependent nucleotide synthesis and an increased glucose shunting into the pentose phosphate pathway for the synthesis of carbon sugars (Montel-Hagen et al., 2009). Therefore, glucose uptake and the balance between mitochondrial and non-mitochondrial glucose metabolism affects erythroid differentiation (Oburoglu et al., 2014).

Moreover, there is evidence that links the CNS pathways modulated by IL-1 with anemia. It has been shown that the replacement of GH was related with a significant rise of Hb, Hct, and number of red cells (Christ et al., 1997). Additionally, experimental *in vitro* and *in vivo* data revealed that IGF can induce proliferation and differentiation of both late and early erythroid progenitors. Consistently, some observational clinical studies demonstrated an inverse correlation of blood levels of IGF-1 with Hb and in various populations (elderly, adult, and dialyzed patients) (Iglesias et al., 1998; Nilsson-Ehle et al., 2005; Succurro et al., 2011). A proof of the direct action of IGF in improving anemia is demonstrated by the association of an IGF genetic polymorphism (related to low IGF concentration) with low Hb levels in a large cohort of adult individuals (Marini et al., 2017).

TNF-α has also direct effects on hemopoiesis; it is able to impair erythropoiesis and erythroid differentiation *in vivo* and *in vitro*, to induce an increase in the apoptosis of immature erythroblasts and a decrease in mature erythroblasts, and to reduce the responsiveness of erythroid progenitors to EPO (Buck et al., 2009). Moreover, TNF-α is responsible mainly for the metabolic changes in lipid metabolism typical of advanced cancer patients, particularly those with cachexia (Patel and Patel, 2017). In detail, TNF-α down-regulates the expression and activity of lipoprotein lipase (LPL) that converts circulating triglycerides into free fatty acids (FFA). TNF-α is also able to decrease the adipocyte expression of FFA transporters, in this way blocking the FFA flow inside the adipocytes, to directly reduce the synthesis of enzymes participating to lipogenesis, e.g., acetyl-CoA carboxylase, Acyl-CoA synthase, and fatty acid synthase, and to induce lipolysis (Carbó et al., 1994). TNF-α has been also involved in the occurrence of insulin resistance through the rise of FFA levels, inhibition of the insulin receptor and insulin receptor substrate-1 (IRS-1) production and induction of IRS-1 Ser/Thr phosphorylation. Among the first transcription factors shown to be targeted negatively by TNF-α signaling in adipocytes was the “adipogenic master regulator,” peroxisome proliferator-activated receptor gamma (PPARγ) that physiologically exert a crucial regulatory action of lipid metabolism (Cawthorn and Sethi, 2008). Downregulation of PPARγ impairs lipid formation and storage in adipose tissue thus leading to a condition known as lipoatrophy (Bing et al., 2006). Notably, a role of PPARγ has been also demonstrated in the regulation of maturation of erythroid progenitors (Nagasawa et al., 2005).

As regard IL-6, it is greatly involved in the pathogenesis of CRA by influencing erythropoiesis at different levels. IL-6 is able to impair proliferation of erythroid progenitors and their response to EPO, and change iron metabolism by modulating liver gene expression and hepcidin synthesis, which is in turn responsible for the functional iron deficiency, typical of CRA (Adamson, 2008). Of relevance, it has been demonstrated that an additional mechanism by which IL-6 is able to impair erythropoiesis is the inhibition of Hb synthesis, independently from the hepcidin-iron pathway, as a consequence of impaired mitochondrial function (by decreasing membrane potential/oxidative phosphorylation) in maturing erythroid cells (McCranor et al., 2014). The key role of IL-6 in determining CRA has been demonstrated in several publications: in particular one publication by us provided the earliest demonstration that IL-6, in conjunction with the stage of disease, represented an independent predictor of Hb values in a cohort of ovarian cancer patients (Macciò et al., 2005). Additionally, IL-6 is a main contributor of the severe immune and metabolic alterations that characterize advanced cancer and contribute to the pathogenesis of CRA. Several years ago, using an experimental animal model to reproduce cancer-related syndrome, Strassmann et al. (1992) showed a crucial function for IL-6 in inducing the early onset of cachexia symptoms, which were independent from the rate of tumor growth, and were associated with loss of muscle and adipose tissues not only due to appetite decrease. Indeed, IL-6 concentrations were proportional to the severity of cachexia and the removal of primary tumor was followed by a significant reduction in IL-6 levels. Consistently, the onset of cachexia symptoms was prevented with anti-IL-6 monoclonal antibodies (Strassmann et al., 1992). Additionally, in rat experimental models it has been observed that, as shown for IL-1, IL-6 acts directly in the hypothalamus to induce the release of CRF, mediated by PGE2 (Turnbull et al., 1998) and is able to impair the production of insulin and the metabolism of pancreatic β cells (Sandler et al., 1990). More recently, IL-6 has emerged as the key determinant of muscle mass wasting in advanced cancer patients (Madeddu et al., 2015). In particular, attention has been recently drawn to the IL-6/STAT3-dependent regulation of the PI3K/Akt/mTOR pathway, which is the principal cellular energy sensor and physiologically activates muscle mass growth. The activation of these pathways mediated by IL-6 as well as the associated increased degradation and low availability of amino acids may also contribute to defective erythropoiesis in advanced cancer patients. Indeed, red cell maturation and the synthesis of Hb are dependent on the activation of mTOR signaling consequent to the increased uptake of amino acids. Conversely, when nutrient/amino acid pools are reduced, mTOR activity is reduced and Hb synthesis is inhibited (Chung et al., 2015; Nathan, 2015). Clearly, anorexia, associated with reduced food intake, and insulin-resistance, with impaired glucose metabolism, also contributes to the inhibition of the mTOR pathway. In turn, anemia, defined as a condition of reduced efficient delivery of O_2_ to peripheral tissues, may inhibit mTORC1 signaling, mainly as a consequence of impaired oxidative phosphorylation and reduced ATP synthesis that lead to mTOR inhibition. Furthermore, CRA is distinguished by “functional iron deficiency” with reduced iron levels and subsequent reduced production of heme, which is a main molecule of muscle myoglobin (Madeddu et al., 2015). This also suggests that anemia contributes to muscle wasting.

## Iron Homeostasis Changes in CRA

Normally, plasma iron is maintained at a stable level by the control of its intestinal absorption and storage. Iron homeostasis is guaranteed by an endocrine system that involves hepcidin, a hormone that controls circulating iron levels by acting on the pathways mediating iron uptake, storage, and release. A small part (1–5%) of circulating iron derives from the diet while mostly is recycled from senescent erythrocytes. More than 50% of the body iron in humans is linked to Hb in erythrocytes, and approximately a quarter is stored in hepatocytes and macrophages. In the circulation, iron usually is coupled with transferrin that transports iron mainly to the bone marrow for erythropoiesis. Circulating iron is delivered inside cells (macrophages of the reticulo-endothelial system) by endocytosis of transferrin and is stored in complex with ferritin (Ganz and Nemeth, 2015). Since erythrocytes hold a greater amount of iron in comparison to other cells, erythropoiesis is particularly sensitive to reduced circulating levels of iron and is inhibited if the concentration of transferrin-bound iron decreases below physiological values. The hampering of erythropoiesis under iron depletion could be useful to convey iron to more critical processes. In fact, differently from erythropoiesis, energy and intermediary metabolism, neurobehavioral activities and immune defense are unaffected by the sequel of iron limitation, unless this gets extremely serious.

**FIGURE 1:**
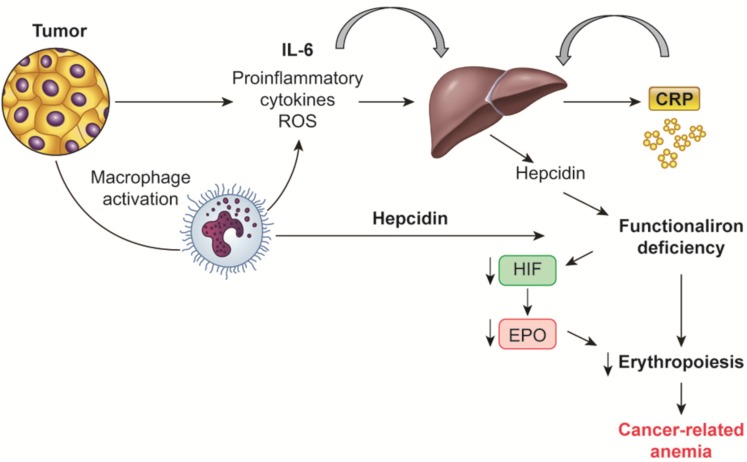
Mechanisms regulating hepcidin synthesis in cancer-related anemia. Tumor and macrophage-derived proinflammatory cytokines, mainly IL-6, induce the liver synthesis of hepcidin, which in turn is responsible of the “functional iron deficiency” typical of cancer-related anemia. Also macrophages by themselves are able to synthesized hepcidin. IL, Interleukin; CRP, C-reactive protein; HIF, hypoxia inducible factor; EPO, erythropoietin.

Dietary iron is absorbed mainly from duodenal enterocytes, where it is transferred from the luminal to the vascular basolateral cell membrane, and released into the circulation by ferroportin. Ferroportin is additionally involved in the iron transport from the storage sites (splenic cells, hepatocytes, bone marrow-derived and tissue macrophages) to the blood. Macrophages are the key cell type that recycles iron from Hb through erythrophagocytosis of senescent cells; macrophages modulate the majority of iron in the body. Iron recycling, storage, and export are tightly regulated by hepcidin. Hepcidin, typically produced by the liver and probably by macrophages, is bound to ferroportin and induces its endocytosis, in this way inhibiting the iron export to the blood and sequestrating iron in duodenal enterocytes and in macrophages, thus limiting erythropoiesis (**Figure 1**). Physiologically, hepcidin production is regulated by a classical positive feedback mechanism through high plasma iron levels, and negatively by low plasma iron levels, hypoxia, and increased erythropoiesis (Ganz and Nemeth, 2015). More recently, erythroferrone has been described as an additional regulator of hepcidin: it is synthesized by erythroblasts stimulated by EPO and inhibit hepcidin production, this in turn enables export of intracellular iron and intestinal iron absorption, thus allowing erythropoiesis (Kautz et al., 2014).

Notably, CRA along with anemia associated with chronic inflammatory diseases are characterized specifically by alterations of iron homeostasis; there is increased iron storage in reticulo-endothelial macrophages and limited iron availability for erythroid progenitors with a subsequent decrease of erythropoiesis. Increased intracellular iron influence erythropoiesis also through the promotion of HIF1α degradation and consequent inhibition of EPO synthesis (Kling et al., 1996).

This condition is called “functional-iron deficiency” and it is correlated with normal or even raised iron reserves in the bone marrow, increased ferritin levels and iron-binding capacity, and normal or decreased serum iron levels (Bron et al., 2001). These peculiar changes have been attributed to the increased synthesis of hepcidin from hepatocytes induced by inflammatory cytokines, mainly IL-6 (Alvarez-Hernandez et al., 1989; Torti and Torti, 2002). In mice, transgenic or constitutive hepcidin overexpression leads to severe anemia associated with low iron availability, while inflammation in animals without hepcidin expression does not determine iron deficiency (Nicolas et al., 2002). This finding suggests that, in chronic inflammatory conditions, hepcidin has a notable function in iron traffic diversion by blocking duodenal iron absorbance as well as iron release by macrophages (Laftah et al., 2004; Nemeth et al., 2004).

In advanced cancer as well as in chronic inflammatory diseases, increase in hepatic production of hepcidin is induced by high levels of IL-6. Increased hepcidin degrades cellular ferroportin, blocks iron excretion and thus increases iron storage in hepatocytes, intestinal enterocytes and macrophages; as a result, less iron is delivered to plasma transferrin (Ludwiczeck et al., 2003). As a consequence, there is restricted iron accessibility for heme synthesis thus inhibiting replication and maturation of erythroid precursors.

Macrophages are the main cell type involved in these events; activated by necrosis, and/or specific chemokines produced by tumors, macrophages consume large quantity of iron by phagocytosis of senescent or injured cells (Schmidt, 2015). Macrophages that exert these actions are called M1 macrophages. Indeed, M1 macrophages, which exert proinflammatory activity associated with high expression of proinflammatory cytokines and ROS, are programmed in an iron-retention mode that promotes their antimicrobial and antitumoral actions. It is well known that activation and polarization of macrophages are closely linked to iron homeostasis: in particular, M1 polarized macrophages are characterized by high expression of hepcidin, high ferritin, low levels of transferrin receptor and ferroportin with consequent blockade of iron release and increased iron storage (Recalcati et al., 2012). During bacterial infection, increased iron removal and sequestering by macrophages looks like to have a double role; first to limit iron availability to exogenous micro-organisms and second, to protect the body from the dangerous consequences of elevated iron levels (and iron-derived highly reactive free radicals), which could result from the tissue injury. Considering that the majority of microorganism depend on exogenous iron for survival, it has been assumed that the above changes in iron metabolism have a host defense function (Ganz and Nemeth, 2015). At the same time, by increasing iron retention activated M1 polarized macrophages may contribute to induce anemia associated to chronic inflammation. At this regard, recently we revealed a strong association between M1 polarized tumor associated macrophages and CRA in a population of ovarian cancer patients (Madeddu et al., 2018). We found also that TAM M1 polarization was associated with high levels of hepcidin and IL-6 as well as with a peculiar shift in iron metabolism-related pathways characterized by high ferritin and low free iron levels both in ascites and in serum. Notably, in the same paper (Madeddu et al., 2018) we demonstrated that M1 TAMs were able *in vitro* to release hepcidin and that IL-6 is was the main responsible of macrophage polarization into M1 phenotype. In fact, we found that IL-6 blockade with a specific anti-IL-6 mAb inhibited this polarization and the consequent synthesis of hepcidin. Consistently with the above evidence, in the clinical setting, it has been widely observed that patients with CRA as well as anemia of chronic inflammatory disease have elevated hepcidin values (Basseri et al., 2012; Shu et al., 2014). Recently, an observational prospective study of our group in a wide sample of patients with advanced cancer at different sites strongly confirmed that Hb value was negatively correlated with hepcidin values, which, in turn, were positively correlated notably with IL-6 as well as with and ferritin, and negatively with serum iron and transferrin saturation values.

## Relationship Between Nutritional Status and Cancer-Related Anemia

During the evolution of the cancer disease, patient nutritional status is severely compromised by symptoms and signs including weight loss associated with significant reduction of muscle mass, increased resting energy expenditure, anorexia, nausea and vomiting. A recent large observational study supported the central role of weight loss, together with the body mass index (BMI), as a negative prognostic factor in cancer patients at diagnosis independently of other more standardized parameters such as tumor site, stage, and PS. Even cancer patients who exhibit at diagnosis a slight decrease of body weight >2.4% have an increased risk of morbidity and mortality (Martin et al., 2015). Loss of body weight represents the main measure for the diagnosis of cancer cachexia (Fearon et al., 2011), which is a complex inflammatory-driven syndrome often accompanying the advanced stages of the neoplastic disease. The weight loss observed in cachectic cancer patients cannot be justified only by the reduction of the supply of nutrients, caused by anorexia and consequent reduced food intake. Energy metabolism changes, which account for a significant increase in resting metabolic expenditure, significantly contribute to loss of body weight (Friesen et al., 2015). Notably, cancer-associated chronic inflammation concurs to the derangements of energy metabolic pathways inflammatory by modulating glucose metabolism, regulating the functioning lipoprotein lipase, which controls the uptake of circulating triglycerides into adipocytes, and changing protein synthesis and degradation, with subsequent depletion in lean body mass (Madeddu et al., 2015). In fact, in cancer patient muscle protein production is decreased while proteolysis is increased, following the raised activity of proteolytic enzymes. Such metabolic behavior is completely different in the liver, where, despite a stable or reduced albumin synthesis, the synthesis of other proteins, especially those of acute phase inflammation (PCR, fibrinogen and hepcidin), is significantly increased (Porporato, 2016). These peculiar changes can also account for the decrease in albumin levels typically associated with high inflammation that affects the Glasgow prognostic score (GPS), an inflammatory/nutritional status based score value, which is closely linked to the worsening of CRA, as demonstrated by our group (Macciò et al., 2015c). Consistently, it is widely demonstrated that malnutrition, in conjunction with weight loss and reduced food intake, is correlated with anemia in patients with chronic inflammatory disease (Hung et al., 2005). This indicates that, along with inflammation, a main contribution to CRA etiopathogenesis is exerted by the nutritional status. In fact, anemia associated with chronic inflammation in advanced cancer patients is not an isolated symptom, but it is more typically associated with weight loss and remodeling of energy metabolism caused by cancer itself (Macciò et al., 2012). Therefore, we hypothesize that the correction of CRA may be achieved more efficiently with a multifactorial therapy that additionally includes treatment of malnutrition.

**FIGURE 2:**
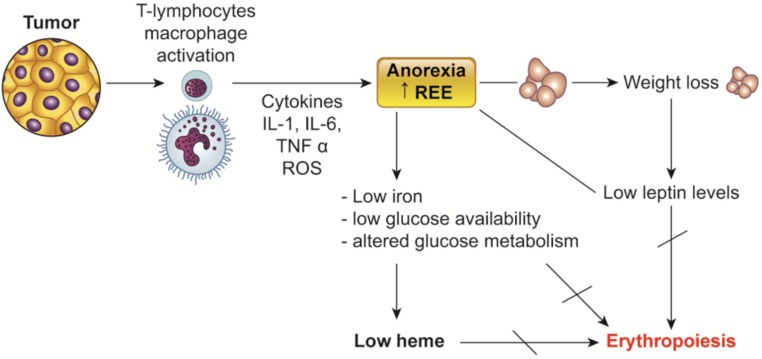
Relationship between nutritional status, leptina and cancer-related anemia. Impaired energy metabolism and low nutrients availability induced by cancer-associated chronic inflammation are involved in the impairment of erythropoiesis. Low iron as well as low heme synthesis as a consequence of altered efficiency of glucose metabolism impair erythropoiesis. Leptin, which decreases as a consequence of such metabolic/nutritional changes, can also influence erythropoiesis by itself. REE, resting energy expenditure.

Consistently with this evidence we found an association between anemia and leptin, which is one of the main marker of the nutritional and metabolic status (Abella et al., 2017), and inversely related to the level of proinflammatory cytokines and stage in cancer patients (Mantovani et al., 2000, 2001; Macciò et al., 2009). In 2005 for the first time in the literature a study from our group showed that the lowest levels of Hb correlated with the lowest values of leptin in ovarian cancer patients. More recently, in a large prospective observational study including patients with different cancer types (Macciò et al., 2015c), we found that leptin, alongside with albumin, cholesterol, and BMI, was positively correlated with Hb. Of relevance, in the same cohort of patients, leptin, in addition to IL-6 and stage, was a predictive variable of Hb. Consistently, leptin values were found to be a predictor of EPO responsiveness in people with kidney failure with anemia (Axelsson et al., 2005; Hung et al., 2005). Noteworthy, it has been also demonstrated that leptin can influence erythropoiesis and stimulate human erythroid progenitor development *in vitro* (Umemoto et al., 1997). However, because anemia in cancer patients is related to the nutritional state that affects the levels of iron, vitamins and other micronutrients useful for erythropoiesis, it is not surprising that a sensitive nutritional marker such as leptin is correlated with Hb levels (**Figure 2**).

## Cancer-Related Anemia Therapy

Cancer-related anemia, not associated with concomitant antineoplastic treatment, is underestimated and undertreated. The rationale to treat CRA would be twofold:

(1) CRA is associated with and aggravates multiorgan failure associated with advanced cancer and characterized by a variety of symptoms. Each of these symptoms can, by themselves, compromise patient quality of life;
(2) CRA has a negative prognostic significance.

The approach to treating CRA must begin with an exhaustive assessment of its defining parameters and identifying its multiple potentially treatable causes: because CRA is mostly multifactorial it should be effectively treated with multitargeted therapies.

It should be underlined that CRA reflects the progressive growth of the underlying disease, thus the eradication of cancer should be the only definitive treatment of this particular form of anemia. However, as the neoplastic disease, in many cases, is not curable, the therapeutic strategies against CRA should target the multiple causes that trigger the disease and should include erythropoietic agents, iron supplementation or blood transfusions, nutritional supplementation, and anti-inflammatory therapies (Weiss and Goodnough, 2005). Future strategies could include chelate-iron therapy, use of hepcidin antagonists and cytokines or hormones that can modulate erythropoiesis under severe inflammatory conditions.

Additionally, if we consider that CRA is also associated with significant impairment of patients’ functional status and QL with symptoms, such as fatigue, that in turn have themselves a multifactorial basis, the evaluation of the outcomes of CRA treatment is complex and cannot be limited to just the increase of Hb levels. In this regard, it is very important to define both the endpoints of CRA treatment and the target level of hemoglobin to obtain the best therapeutic outcome in terms of improvement of patient’s symptoms. The last “Update Committee of the American Society of Hematology/American Society of Clinical Oncology (ASCO/ASH)” published in 2010 (Rizzo et al., 2010) recommended that: “The first objective to achieve is to reduce blood transfusion requirements. The Hb target should be the lowest concentration needed to avoid transfusions, which may vary by patient and condition.” Similarly, the NCCN guidelines stated that “elimination of symptoms and avoidance of transfusions are the main goals of ESA therapy” (Rodgers et al., 2017).

It should be underlined that during erythropoiesis, homeostatic mechanisms compensate for increased EPO synthesis when Hb drop to a value lower than 12 g/dl (Finch, 1982). Notably, the target Hb value to prevent the necessity of blood transfusions, should be at least ≥ 10 g/dl, but it has been demonstrated that the highest improvement of QL is achieved at Hb values of 12 g/dl, thus indicating the relevance of keeping Hb values within this range (Crawford et al., 2002). More precisely, data from QL assessment related to the amelioration of anemia show that an optimal improvement is achieved between Hb 11 gr/dl and 12 gr/dl.

## Transfusion of Concentrated Erythrocytes

Red blood cells (RBC) transfusions are almost universally successful in raising Hb levels and the oxygen transport capacity of the blood. Therefore, they represent a fast and effective therapeutic intervention useful to ameliorate rapidly the patient’s symptoms, e.g., breathlessness, and improve health-related QL (Rodgers et al., 2017). Prior to rHuEPO being available, blood transfusions were the only therapeutic option for the improvement of symptomatic anemia in cancer patients. The major benefit, not provided by any alternative therapy for anemia, is the fast Hb and Hct value rise (Bohlius et al., 2006). RBC transfusions are particularly useful in case of severe symptomatic anemia or anemia compromising patient life (Hb <7–8 g/dl). These thresholds cannot be applicated to people affected by acute coronary syndrome and with complications at risk of bleeding, where blood transfusion may be indicated also for Hb values ≥ 8 g/dl (Carson et al., 2016). The need for blood transfusion depends other factors such as age, compromised vital signs and severe tissue hyperoxygenation (Simon et al., 1998). Notably, the last published NCCN guidelines (Rodgers et al., 2017) indicate that RBC transfusions should be considered not on the basis of a specific threshold value of Hb but in patients with symptomatic anemia, in high risk patients (e.g., those undergoing high-dose chemo-or radiotherapy with cumulative decrease of Hb levels) or asymptomatic patients with comorbidities (e.g., heart disease, COPD, cerebral vascular disease). Undoubtedly, patients who are positive for the presence of multiple alloantibodies and those with specific religious beliefs are unable to receive blood transfusions. In advanced cancer patients imminent to surgery, blood transfusions are routinely employed to treat pre-operative anemia and minimize blood loss related to surgical procedures. Perioperative blood management strategies such as pre-surgery autologous blood donation, acute normovolemic blood dilution, intraoperative cell collection, rHuEPO therapy, optimal hemostasis, mini-invasive surgeries may minimize the requirement of allogeneic blood transfusion in case of scheduled surgical procedures (Weber et al., 2008). Some studies evaluating the clinical effect of transfusions showed a survival benefit in neoplastic patients receiving transfusions (Grogan et al., 1999; Kader et al., 2007). In particular, in the study by Grogan et al. (1999), patients that reached a specific Hb value with blood transfusion showed a survival rate similar to patients that had that Hb value spontaneously; then, blood transfusion appeared to reduce the negative prognostic role of low baseline Hb. Additionally, blood transfusions are beneficial in terms of patient subjective symptoms such as breathlessness (Gleason and Spencer, 1995; Mercadante et al., 2009) and fatigue (Gleason and Spencer, 1995; Tanneberger et al., 2004; Mercadante et al., 2009; Brown et al., 2010). However, blood transfusions have relevant acute and long-term risks, which include fever, transfusion-related and potentially fatal allergic reactions, transmission of infectious diseases, alloimmunization, iron overload and immunosuppression with potential favoring effect on cancer progression (Goodnough, 2005; Toy et al., 2005). The risk of iron overload increases in patients that require frequent transfusions: excessive iron is dumped in critical part of the body such as the heart and the liver causing cumulative toxicity. Moreover, blood transfusions have been correlated with more thromboembolic events and related death in hospitalized neoplastic patients (Khorana et al., 2008). In 2012, a systematic review that evaluated the benefit and risks of blood transfusion in very advanced cancer patients showed a significant increase of Hb levels and a subjective symptomatic response rate, especially in terms of fatigue and dyspnea. Patient survival varied greatly ranging between two and 293 days and a high percentage of patients (23–35%) were dead after only 2 weeks from transfusion. The review concluded that high-quality studies are warranted to establish the risks and effectiveness of blood transfusion in advanced cancer patients and to determine patients who are candidate to obtain a benefit (Preston et al., 2012). Notably, the concern of the premature death after treatment for CRA in very advanced cancer patients raises the question of whether anemia treatment may be causative to the increased risk of death.

## Treatment with rHuEpo

Recombinant HuEPO received its first approval for the therapy of anemia in patients with chronic kidney failure. In 1993 FDA approved r-HuEPO also for the therapy of anemia in cancer patients. Currently different short-and long-acting formulations of rHuEPO are available: r-HuEPOα, r-HuEPOβ, and darbopoetin α. Because of the glucidic component, r-HuEPO has a longer half-life after subcutaneous administration compared to natural EPO that has a half-life of 8.5 h (24 h for r-HuEPOα and 20.5 h for r-HuEPOβ) (Storring et al., 1998). As short-acting drugs, first generation rHuEPO necessitated repeated doses to keep appropriate Hb levels. Conversely, darbopoetin-α is a modified hyperglycosylated epoetin and has a longer half-life after its subcutaneously administration (about 49 h): this allows to increase the interval of the dose schedule necessary for Hb level maintenance (Bohlius et al., 2006). Recombinant HuEPOs are biologically and pharmacologically active intravenously, intraperitoneally but especially subcutaneously (s.c.). All three recombinant erythropoietin proteins have similar efficacy (Halstenson et al., 1991; Storring et al., 1998). The standard therapeutic regimen is 150 IU/kg three times a week for r-HuEPOα, or 40,000 IU once weekly for r-HuEPOα, or 30,000 IU once weekly for r-HuEPOβ. The recommended dose of darbopoietin α is 2.25 μgr/kg weekly or 500 μgr every 3 weeks.

More recently, several biosimilar EPOs have been developed and introduced in the clinical practice: biosimilar epoetin alfa (e.g., Binocrit^®^, Sandoz) and epoetin zeta (e.g., Retacrit^®^, Hospira). A biosimilar is a biological drug, which is highly superimposable as for primary structure, mechanism of action, and treatment target, to the approved reference biological drug. The great degree of similarity is revealed by the results of an extensive clinical comparison of pharmacokinetics and pharmacodynamics properties by regulatory authorities. The approvals of biosimilar epoetin alfa and zeta in 2007 were founded on strong assessments including molecular characteristics, preclinical *in vivo* and *in vitro* experiments in animals, clinical trials in the target populations, and pharmacovigilance surveillance studies (Jelkmann, 2010). The safety data indicate that adverse effects among patients treated with epoetin zeta (Retacrit^®^) are consistent with those known for epoetin α (Michallet and Losem, 2016). Similarly, the majority of safety data for biosimilar epoetin alpha (Binocrit^®^) are favorable. However, one confirmed and one suspected pure red cell aplasia (PRCA) have been reported during a clinical trial in patients receiving the subcutaneous formulation (Haag-Weber et al., 2012). As a result, biosimilar epoetin alpha is now only accessible for intravenous administration, while epoetin zeta is presented in both intravenous and subcutaneous formulation.

### Efficacy of rHuEPO for the Management of CRA

Randomized clinical studies have showed that rHuEPO increased Hb values and reduced the number of hemotransfusion in anemic cancer patients (Ludwig et al., 2009). In particular, regarding CRA, a phase II, double-blind, placebo-controlled trial demonstrated that more patients in the arm receiving darbopoetin-α achieved the hematopoietic response and the target Hb levels in comparison to the placebo arm (Gordon et al., 2008). Another phase III randomized double-blind trial carried out in advanced lung cancer patients demonstrated that treatment with epoetin-α was associated with fewer transfusions and achieved a higher Hb rise than placebo (Wright et al., 2007) The ability of rHuEPO to achieve a significant reduction of RBC transfusions and a higher hematopoietic response was supported by a large Cochrane meta-analysis, which analyzed a total number of 20,102 patients from 91 randomized clinical studies, which evaluated ESAs for the therapy of anemia in cancer patients either undergoing or not undergoing concomitant chemotherapy (Tonia et al., 2012). Moreover, there is a strong evidence that rHuEPO may improve QL, fatigue and other specific anemia-related symptoms (such as dizziness, chest discomfort, headache, trouble walking) (Tonia et al., 2012). A number of open-label non-randomized and community-based trials in advanced cancer patients with CRA demonstrated that a progressive amelioration of QL obtained by ESAs was significantly correlated with the increased Hb levels (Quirt et al., 2001; Bogdanos et al., 2004; Mystakidou et al., 2005; Wright et al., 2007; Smith et al., 2008). In particular, Crawford et al found that the highest improvement of QL was found if Hb value rises from 11.0 to 12.0 g/dl and ≥1 g/dl (Crawford et al., 2002). In 2014, a systematic meta-analysis confirmed that ESAs determine a clinical significant amelioration of anemia-related symptoms (evaluated by FACT-An), while the improvement in fatigue-related symptoms (FACT-F), did not obtain the value needed for a clinically significant change (Bohlius et al., 2014). These results indicate that fatigue, in advanced cancer patients is influenced by several parameters other than anemia. Therefore, the mere amelioration of Hb levels could not have appropriately counteracted the multifactorial pathophysiology and psychological aspects of fatigue in advanced cancer patients. Otherwise ESAs may have additional beneficial effect other than the correction of anemia, such as neuroprotective, anti-inflammatory, vascular and metabolic actions, directly related to the action of EPO on other target organs such as central/peripheral nervous system and heart (Nekoui and Blaise, 2017).

## Adverse Effects of Esas

Both randomized clinical studies and systematic reviews demonstrated a significantly higher risk of thromboembolic events in patients receiving ESAs for CRA (Wright et al., 2007; Bennett et al., 2008; Smith et al., 2008; Tonia et al., 2012). Excessively high Hb levels at baseline prior to treatment with rHuEPO were related with a significant increased occurrence of thromboembolism in some trials in cancer patients with anemia undergoing chemotherapy (Aapro et al., 2009; Spivak et al., 2009). A survey including five trials by the Agency for Healthcare Research and Quality concluded that there was a trend toward fewer thromboembolic events if ESA administration was started only at Hb < 10 g/dl (Grant et al., 2013). Conversely, in 2012 a Cochrane metaanalysis of 91 controlled studies on ESAs therapy for cancer anemia documented a significantly elevated risk of thrombotic events regardless of the baseline Hb levels (Tonia et al., 2012). As regard the relationship between target Hb values and the frequency of thromboembolism and vascular accidents in end-stage renal disease (ESRD) patients receiving rHuEPO (Singh et al., 2006), it should be remarked that in some of the trials reporting an higher risk of thromboembolism and related mortality, Hb target levels were above standard, ranging from 13 to 15 g/dl (Henke et al., 2003; Leyland–Jones et al., 2005; Juneja et al., 2008). In a large trial evaluating the use of darbepoetin-α for CRA in patients not undergoing concomitant antineoplastic treatment, thromboembolism was not more frequent in people with Hb > 13 g/dl, nor with Hb rise > 1 g/dl in 2 weeks compared to those who did not (Smith et al., 2008). Moreover, it should be highlighted that no randomized clinical trial to date have evaluated any prophilactic approach to counteract the incidence of thromboembolism by administering, for example, anti-coagulant agents especially in the setting of advanced cancer patients, which present by themselves an elevated risk of thromboembolism.

Other adverse events associated with the use of ESAs, such as hypertension, thrombocytopenia/hemorrhage and seizures have been reported. The Cochrane meta-analysis by Tonia et al. (2012) reported and increased risk ratio of hypertension by approximately 30% in cancer patients receiving ESAs. The same Cochrane (Tonia et al., 2012) showed a significant increased risk to develop thrombocytopenia in erythropoietin-treated cancer patients. Some case of seizures have been associated to ESA treatment in cancer patients while the incidence was not significantly different in comparison to control arm (Tonia et al., 2012).

## Safety Issues and Concerns in Clinical Practice

In 2007 FDA published a warning statement limiting the indication of ESAs only for the therapy of chemotherapy-induced anemia and discouraging their administration when the antineoplastic therapy is finished (Goldberg and Goldberg, 2008; Hagerty, 2008); FDA also indicated that ESAs should be used only when the antineoplastic treatment has a palliative intent and clarified there is not a upper range for target hemoglobin but that the objective of ESA therapy should be the lowest Hb value to avoid transfusion (Goldberg and Goldberg, 2008). Such alerts were based on the results of several clinical trials that found that ESAs treatment in cancer patients was associated both with an inferior survival and worse cancer outcomes (Newland and Black, 2008; Bohlius et al., 2009; Pfeffer et al., 2009; Tsuboi et al., 2009): this has been correlated with an excess of thromboembolic events correlated with the levels of Hb reached (Tonia et al., 2012). Additionally, it was also hypothesized that the higher mortality was dependent on EPO ability to stimulate tumor growth (Henke et al., 2006; Brown et al., 2007). However, more recently, in 2009, a pooled analysis, involving more than 13,000 cancer patients, failed to confirm the data about the increased mortality risk associated with ESA (Bohlius et al., 2009).

Following the above concerns, the guidelines from the American Society of Clinical Oncology (ASCO) and American Society of Hematology (ASH) (Rizzo et al., 2010) as well as from the National Comprehensive Cancer Network (NCCN) for the appropriate therapy of anemia of cancer patients undergoing antiblastic therapy recommended considering the utilization of ESAs for selected patients according to FDA indications. In detail, they stated that ESA may be indicated to for patients with chemotherapy-induced anemia with Hb < 10 g/dL to decrease transfusion. Clinicians should start treatment after having discussed with patients the potential harms (e.g., thromboembolism, shorter survival) and benefits (e.g., decreased transfusions) in comparison to the potential harms (e.g., serious infections, immune-mediated adverse reactions) and benefits (e.g., rapid Hb improvement) of transfusions. The Committee cautions against ESA use under other circumstances, in particular in patients who are not receiving concurrent myelosuppressive chemotherapy. As regard the FDA label that limit the indication for ESA to patients receiving chemotherapy for palliative intent, Rizzo et al. (2010) did not include this point. Indeed, to date no study or meta-analysis has analyzed the outcomes of ESA therapy by dividing patients according to chemotherapy aim. In 2012 a Cochrane meta-analysis assessing 91 trials with 20,102 patients found that if ESAs are used correctly for the therapy of chemotherapy-induced anemia as well CRA only if Hb is below 12 g/dl, no increase in overall mortality and on-study mortality have been observed (Tonia et al., 2012).

In clinical practice, cautions should be used when using ESA concomitant with chemotherapy regimen and cancers associated with increased risk of thromboembolism. Moreover, ESAs are contraindicated in patients with uncontrolled hypertension.

## Iron Supplementation

Cancer-related anemia is characterized by both a defective incorporation of iron into developing erythrocytes and by erythropoietin deficiency. Therefore, it has been hypothesized that treatment of CRA could include a combination of ESAs and iron supplementation.

Iron formulation assessed in cancer patients include oral and parenteral forms (low-molecular weight iron dextran, ferric gluconate and iron sucrose.) To date, in a recent systematic meta-analysis (Mhaskar et al., 2016) the addition of iron to ESAs versus ESAs alone for chemotherapy-induced anemia, showed to be associated with increased hematopoietic response, lowered RBC transfusions, and improved Hb changes. The meta-analysis did not show any benefit in time to hematopoietic response nor any improvement in QL in patients supplemented with iron plus ESAs in comparison to ESAs alone. No treatment-related deaths have been reported (Mhaskar et al., 2016). Various routes of iron administration are available: oral intramuscular or intravenously. The bivalent (ferrous) form of oral iron has better bioavailability that the trivalent one (ferric). As for safety, ferrous gluconate is safer than iron dextran (Fishbane and Kowalski, 2000). As for tolerability, parenteral iron may be associated with nausea, vomiting and/or diarrhea, hypotension, pain, hypertension, dyspnea, pruritus, headache, and dizziness. The majority of adverse events in literature were associated with the use of high molecular weight iron dextran, which is anymore recommended and has been today replaced in the clinical practice by the other formulations (Baribeault and Auerbach, 2011; Rodgers et al., 2017).

Nevertheless, the use of iron for CRA is controversial. In fact, in addition to an absolute iron deficiency due to reduced nutritional intake or blood loss, these patients could present an iron functional deficiency with reduced saturation of transferrin and high levels of ferritin, which develops as a consequence of the chronic inflammatory status. This condition is characterized by an increase in iron storage with a limited iron availability or erythropoiesis. Moreover, it is characterized by a compromised iron intestine absorbance system mediated by hepcidin. In particular, this last piece of evidence suggested that cancer patients with anemia should not have any benefit from oral iron formulation, but they should receive intravenous iron. Several studies have compared intravenous versus oral iron administration in combination with ESAs for chemotherapy-induced anemia. The majority of these trials demonstrated that intravenously iron supplementation (in comparison to either orally administered iron or no iron) increases Hb response to ESAs and decreases the number of transfusions (Auerbach et al., 2004; Henry et al., 2007; Bastit et al., 2008; Pedrazzoli et al., 2008). A recent meta-analysis relevant to this issue (Mhaskar et al., 2016) confirmed a superior efficacy of intravenous iron combined with ESAs in comparison to oral iron regarding hematopoietic response, need of RBC transfusions, and improvement in Hb.

Noteworthy, the largest clinical trial performed to date by Steensma et al. (2011) demonstrated the lack of benefit of adding intravenous iron to ESAs in anemic cancer patients with signs of functional iron deficiency. These controversial results may be at least in part attributed to the fact that the clinical trials assessing the effectiveness of the combination of ESAs with orally or intravenously administered iron for chemotherapy-induced anemia, incorporated an heterogeneous population, including both patients in early and advanced stages, where the pathogenesis of anemia and the alterations of iron metabolism are completely different. In this regard, in 2010 we performed a randomized, prospective trial in a selected population of advanced cancer patients with chronic inflammation, which have CRA yet before starting chemotherapy (Macciò et al., 2010). The study had the scope to assess whether the supplementation with orally administered lactoferrin compared to intravenous iron, both added to rHuEPO, was effective and safe, for the therapy of CRA in a cohort of 148 advanced neoplastic patients receiving chemotherapy. Lactoferrin is an iron-binding protein with a molecular weight of 80 kD belonging to the transferrin family, which may play a relevant antimicrobial and antiinflammatory activity in the immune response. Enrolled patients were randomized to receive intravenous ferric gluconate (125 mg/week) or lactoferrin (200 mg/day), each combined with s.c. rHuEPOβ, 30,000 UI/week, for 12 weeks. Hemoglobin increased significantly in the two arms; both arms obtained a similar Hb improvement, hematopoietic response, time to hematopoietic response or mean change in serum iron, CRP, or erythrocyte sedimentation rate. Notably, ferritin levels lowered in the lactoferrin, while they augmented in the intravenous iron arm. Therefore, the results of this trial showed that oral lactoferrin was effective as intravenous iron in terms of Hb increase. The decrease of ferritin in the lactoferrin arm probably suggests that ferritin is better able to modulate iron metabolism and ameliorate iron recycling.

On the basis of currently available data, patients with CRA should receive iron supplementation, especially when they are not responsive to the treatment with erythropoietic agents alone. However, martial therapy could not be recommended for patients with functional iron-deficiency and high levels of ferritin. In this case, iron overload can favor ROS synthesis thus aggravating the oxidative stress status leading to subsequent endothelial damage and increased risk of cardiovascular complications.

## Combined Multitargeted Approach of CRA

Considering the multifactorial mechanisms leading to CRA, mainly attributable to the condition of chronic inflammation that characterizes advanced cancer patients, some combined targeted approaches have shown a significant benefit in ameliorating anemia and related symptoms. Moreover, not all patients with CRA benefit from the use of ESA alone: up to 15–20% still require RBC transfusions, and only 50–70% have an Hb increment of ≥1 g/dl after administration of ESA for 8–12 weeks (Beguin, 1998). Evidence from several studies in patients with end-stage renal disease and anemia showed that ESAs hyporesponsiveness may be due to several factors: a weakened iron supplying to developing erythroid cells due to functional iron deficiency (Tarng et al., 1999); increased amount of erythropoiesis-inhibiting inflammatory cytokines (Ribeiro et al., 2016); reduced energy and nutrients supply due to the compromised food intake and metabolic changes (Bistrian and Carey, 2000). A multitargeted approach may be useful in circumvent these events and improve the effectiveness of ESAs.

In this regard, a randomized clinical trial (Daneryd et al., 1998) in 108 cancer patients with advanced tumors at different sites with cachexia not undergoing concomitant chemotherapy showed that the administration of rHuEPO in association with the anti-inflammatory agent indomethacin achieved a higher increase in Hb levels as well as increased oxygen uptake, and respiratory activity with a better-preserved exercise capacity in comparison to the arm with indomethacin alone. These results were correlated with a significant reduction in inflammatory markers in the experimental arm. These results have been confirmed in further analysis published by the same authors where the combined approach with indomethacin and rHuEPO showed to improve Hb levels and functional capacity; however, it did not improve significantly subjective measures of QL (Lindholm et al., 2004). Also another randomized study by Lundholm et al. (2004), evaluated whether the addition of a specialized, nutritional support to a combined treatment with indomethacin and rHuEPO, and confirmed that the combined approach prevented CRA and improved significantly the energy metabolism and functional outcome in advanced cancer patients with cachexia. Indeed, indomethacin by reducing cytokine production and inflammation, may improve by itself anemia (Nieken et al., 1995) and increase responsiveness to EPO treatment (Bistrian and Carey, 2000). These properties are common to other NSAIDs that may thus obtain similar results such as aspirin and COX-2 inhibitor.

At this regard, some papers from our group demonstrated that a combined approach for the therapy of metabolic/inflammatory changes and associated symptoms (cachexia) related to advanced cancer was able to significantly counteract CRA. In detail, two case reports published by our group confirmed that a supportive multitargeted anticachectic approach including L-carnitin, curcumin, lactoferrin, rHuEPO and the COX-2 inhibitor celecoxib was able to counteract CRA in a patient with metastatic hormone-refractory prostate cancer (Macciò et al., 2015a) and in a patient with myelofibrosis with associated cachexia (Macciò et al., 2015b), respectively. The combined approach in both patients achieved a rise of Hb and serum iron values, and a concomitant decrease of inflammatory markers, ferritin, and hepcidin. The treatment in both patients was associated with an improvement of weight, lean body mass, grip strength, fatigue, and overall QL.

Therefore, when increased risk factors or comorbidities are lacking, the use of rHuEPO associated with the concomitant administration of low molecular weight heparin, for counteracting the well-known prothrombotic status of advanced cancer, plus the modulation of iron metabolism with appropriate supplements, and an adequate nutritional support, is able to improve CRA, associated symptoms and impairment of QL.

## Conclusion

Cancer-related anemia has a high incidence and significant clinical impact on patient prognosis and QL. However, so far the clinical studies testing the application of ESAs have been limited to chemotherapy-related anemia. About this point, it should be remembered that ESAs are indicated for Hb values < 10 g/dl, not considering that many patients (particularly in the advanced stages of disease) have already anemia before the start of chemotherapy, with Hb values equal to or lower than this threshold (Ludwig et al., 2004). Considering the evidence regarding iron-restricted erythropoiesis, several studies found that intravenous iron supplementation had higher efficacy than oral iron administration for the therapy of CRA (Henry, 2010). However, more recently the role of intravenous iron administration, in case of functional iron deficiency typical of CRA, has been disputed (Spivak, 2011). Accordingly, in our randomized clinical study we hypothesized the necessity to select the way of iron supplementation on the basis of patient clinical characteristics. Furthermore, we showed that the selective modulation of iron metabolism through oral lactoferrin, in advanced patients with CRA during antineoplastic treatment, decreases excessive iron storage (ferritin) and reduces ferritin levels, and supported the ESAs activity, comparably to intravenous iron administration (Macciò et al., 2010).

It is crucial to underline that cancer growth and related inflammatory response determines alterations of energy metabolism and feeding (cancer-related anorexia and cachexia), that may potentially induce anemia. Consistently, we demonstrated that parameters of the nutritional and energy status of the patient such as leptin, were positively correlated with Hb levels. Also, we showed that the GPS, a score based on the inflammatory/nutritional status, was predictive of Hb levels (Macciò et al., 2015a). This piece of evidence is strongly in accordance with findings observed in chronic kidney failure patients, where the “malnutrition-inflammation score” showed to be widely correlated with anemia (Kalantar-Zadeh et al., 2001; Molnar et al., 2011; Rattanasompattikul et al., 2013). Until now, the association of the nutritional status with the hemoglobin levels in cancer patients has not been sufficiently assessed. Conversely, in people with chronic kidney failure, where this correlation has been largely found, the role of an adequate nutritional status to improve anemia, particulary in those patients candidate to ESAs, is now strongly recognized (Takeda et al., 2002; Axelsson et al., 2005; Hung et al., 2005).

A proper characterization of cancer patients with anemia on the basis of tumor staging and inflammation/metabolic-related symptoms is thus warranted to identify specific parameters that will enable the design and implementation of the best therapeutic strategy to treat CRA, in which inflammation and metabolic impairments seem to play pivotal roles (Steensma, 2008). In this context, the finding that treatment with anti-IL6 mAb in patients with advanced cancer at different sites is able to significantly increase Hb levels is of high relevance (Bayliss et al., 2011; Coward et al., 2011). In conclusion, on the basis of the evidence discussed here, we can conclude that the comprehension of the multifactorial pathogenetic pathways leading to CRA is pivotal to identify the most adequate and effective treatments. Currently, the leading international scientific societies have developed protocols that have focused primarily on the treatment of chemotherapy-induced anemia. The data discussed herein indicate that an accurate assessment of the neoplastic patient must be achieved prior to start the therapy for CRA; besides ESAs, the approach should provide a specific multitargeted therapy, including, e.g., adequate caloric supplementation, an antioxidant/anti-inflammatory treatment and personalized iron supplementation.

## Author Contributions

CM and AM contributed to the conception of the work, analysis and interpretation of data, drafting of the manuscript, and critical revision of the manuscript. GG, GA, VA, and ES contributed to the analysis and interpretation of data and drafting of the manuscript. RD contributed to the analysis and interpretation of data, and drafting of the manuscript in particular as regard the paragraph “safety issue and concerns in clinical practice.” All the authors approved the final version to be published; all authors agreed to all aspects of the work.

## Conflict of Interest Statement

The authors declare that the research was conducted in the absence of any commercial or financial relationships that could be construed as a potential conflict of interest. The handling Editor co-organized a Research Topic with the authors CM, AM and confirms the absence of any other collaboration.

## Footnotes

1 http://www.who.int/vmnis/indicators/haemoglobin.pdf

2 https://evs.nci.nih.gov/ftp1/CTCAE/About.html
